# Cone beam computed tomographic analysis of maxillary premolars and molars to detect the relationship between periapical and marginal bone loss and mucosal thickness of maxillary sinus

**DOI:** 10.4317/medoral.20587

**Published:** 2015-08-04

**Authors:** Duygu Goller-Bulut, Ahmet-Ercan Sekerci, Emre Köse, Yildiray Sisman

**Affiliations:** 1 DDS. Research Assistant, Department of Oral and Maxillofacial Radiology, Faculty of Dentistry, Erciyes University, Kayseri, Turkey; 2 DDS, PhD. Assistant Professor, Department of Oral and Maxillofacial Radiology, Faculty of Dentistry, Erciyes University, Kayseri, Turkey; 3 PhD. Professor, Department of Oral and Maxillofacial Radiology, Faculty of Dentistry, Erciyes University, Kayseri, Turkey

## Abstract

**Background:**

This study assessed the relationship between mucosal thickness (MT) of the maxillary sinus and periodontal bone loss (PBL) and periapical condition of related teeth. We also aimed to identify the association between root apices and the inferior wall of the maxillary sinus using Cone beam computed tomography (CBCT).

**Material and Methods:**

In this study, CBCT images of 205 patients with 410 maxillary sinuses were examined, retrospectively. A total of 582 maxillary molars and 587 premolars were observed. The relationship of each root with maxillary sinus and apical lesions of these roots were classified, PBL was examined and the situations of adjacent teeth were estimated. The effect of these conditions on sinus mucosal thickness (MT) was evaluated.

**Results:**

There was a significant correlation between MT of maxillary sinus and both PBL and age (r = 0.52, *p*=0.000 and r = 0.111, *p*= 0.002, respectively). The frequency of MT increased as the severity of apical lesion enlarged. A positive correlation was found between MT and degree of PBL and periapical lesions. To reveal the association between MT and pulpoperiapical condition bivariate correlation was done and a significant relationship between the pulpoperiapical condition and MT was found (r = 0.17, *p*=0.000).

**Conclusions:**

This retrospective study showed that MT of the maxillary sinus was common among patients with PBL and MT was significantly associated with PBL and apical lesions. The relationship of maxillary sinus to adjacent teeth had also positive correlation with MT. CBCT imaging enabled better evaluation of maxillary sinus, posterior teeth and surrounding structures compared to other imaging tools.

**Key words:**Maxillary sinus mucosal thickness, apical periodontitis, periodontal bone loss, CBCT.

## Introduction

Maxillary sinuses are pneumatic cavities within maxillary bone that communicate with the nasal cavity by ostium (1,2). The sinus is lined with a thin respiratory mucous membrane referred as the Schneiderian membrane. This membrane adheres to the periosteum and is about 1 mm thick. The normal sinus mucosa is not imagined on a radiograph. Although, when the mucosa comes to be irritated from either an infectious or allergic process, it may increase and may be seen on radiograph. Mucosal thickness (MT) greater than 3 mm is most likely pathological (3). The radiographic image of thickened mucosa is as a non-corticated radiopaque band, paralleling the bony wall of the sinus (3).

Maxillary sinus is very closely linked to the alveolar crest and in some cases the floor can be perforated by the apices of the teeth. This close anatomic proximity of root apices to the maxillary sinuses makes dental disease, especially periapical lesion, a potential source for the spread of the disease into the maxillary sinuses (4). In addition, the close relationship between the maxillary sinus floor and the roots of molars and premolars can lead to accidental oroantral communication (5,6). It is essential for clinicians to be aware of the exact relationship between the apical roots of the maxillary teeth and the maxillary sinus floor. Cone beam computed tomography (CBCT) has raised the interest in revisiting the anatomical features of the jaws. It provides an accurate evaluation of maxillary bone quality and quantity around posterior root apices without the distortion and superimposition caused by teeth and the surrounding structures (7,8).

The aim of the present study was to assess the relationship between sinus mucosal thickness and age, the anatomic position of teeth, degree of periapical lesions and periodontal bone loss using CBCT.

## Material and Methods

The Ethical Committee of the University of Erciyes, Faculty of Dentistry, approved the study protocol that has therefore been performed in accordance with the ethical standards laid down in the 1964 Declaration of Helsinki. Written informed positive consent was obtained from the parents.

The materials for this study were obtained from the Department of Oral and Maxillofacial Radiology, Faculty of Dentistry, Erciyes University. CBCT images had been taken because of the patients previous dentomaxillofacial problems. Cases were enrolled provided the scans showed the bilateral posterior area of the maxilla includes the premolars and molars in both sides.

The inclusion criteria were described as follows:

1- Good quality of the CBCT images.

2- Existence of at least one of the premolars or molars in each left or right sides (fully erupted teeth and fully formed apices).

3- No sign of acute non-odontogenic sinusitis, including air-fluid level and thickening of all the sinus walls.

4- No instruction of CBCT due to trauma or developmental problems.

Cases with the signs of acute sinusitis with air-fluid level and complete mucosal thickness in all the sinus walls were excluded.

- Evaluation of the images

The CBCT images were analyzed in the NNT viewer which is a simple version of the NNT software of the CBCT (Newtom5G, QR, Verona, Italy) machine in a Dell Precision T5400 workstation (Dell, Round Rock, TX, USA), and a 32-in. Dell LCD screen with a resolution of 1,280 × 1,024 pixels in a darkroom. The contrast and brightness of the images were adjusted using the image processing tool in the software to ensure optimal visualization.

Patients with at least one exposed maxillary sinus were included in the study. A total of 205 patients (104 females and 101 males) who had 410 exposed maxillary sinuses met our inclusion criteria. The age of the patients ranged from 16 to 77 years with a mean age of 38.8.

- Evaluation of Periapical Status 

Using the periapical index scoring system (PAI) the periapical position was evaluated as follows.

1. Normal periapical structures;

2. Minor changes in bone structures.

3. Some mineral loss and changes in bone structure.

4. Periodontitis with a well-defined radio lucent area;

5. Severe periodontitis with exacerbating structures (9).

If the tooth had more than one periapical lesion that was associated with roots, the lesion with the most severe pathology was recorded.

- Evaluation of Anatomic Relationship between the Sinus Floor and Associated Teeth

To inspect a probable correlation between the CBCT findings and oroantral communication, the images were inspected. The vertical relationship between the inferior wall of the maxillary sinus and the maxillary premolars and molars was assessed using a modification of Kwak’s classification (10). These vertical relationships were classified into five types based on the CBCT cross- sectional images:

Type I: Buccal and palatal roots apices were not contact with the inferior wall of the maxillary sinus.

Type II: Buccal and palatal roots apices were in contact with the cortical borders of inferior wall of the maxillary sinus.

Type III: Buccal root apices were projecting into the sinus cavity over the inferior wall of the maxillary sinus.

Type IV: Palatal root apices were projecting into the sinus cavity over the inferior wall of the maxillary sinus.

Type V: Buccal and palatal root apices were projecting into the sinus cavity over the inferior wall of the maxillary sinus.

- Evaluation of PBL

PBL was evaluated from the coronal, tangential and sagittal views. The normal condition of the alveolar crest was expected to be 2 mm under the cemento-enamel junction (CEJ) (1,11). To compute the amount of PBL, the distance between the point 2 mm under the CEJ and the upper point of the alveolar bone was measured. We measured the PBL in the mesial and distal sides of the teeth and selected the higher measurement for statistical analysis, which could be more exact. Furthermore, we classified the amount of the PBL as follows:

1. No finding of PBL,

2. Mild, <25% bone loss,

3. Moderate, 25-50% bone loss,

4. Severe, >50% bone loss (1).

- Evaluation of Pulpoperiapical Condition

To evaluate the possible relationship between pulpoperiapical condition and MT, we categorized the existing teeth into five groups (1):

1. Normal,

2. Teeth with prosthetic crown,

3. Teeth with root canal therapy,

4. Teeth with both root canal therapy and crown,

5. Teeth with severe carries that proceed more than half of dentin.

- Evaluation of MT

MT in the maxillary sinus floor was evaluated from the cross-sectional, coronal and tangential views. In each sinus, mucosal thickness was measured in the point of maximum thickness from the sinus floor over the all teeth apices (1,3). MT was considered to be when there was a thickness of >1 mm. The amount of MT was also classified into five class:

1. Class 1. Normal (no mucosal thickness);

2. Class 2. 0-2 mm;

3. Class 3. 2-4 mm;

4. Class 4. 4-10 mm;

5. Class 5.More than 10 mm (3).

- Statistical analysis

All the CBCT images were evaluated by the same oral and maxillofacial radiologist. To assess the intraexaminer calibration, 20% CBCT images were measured again. The intraclass correlation coefficient was 0.95 for PBL measurements and 0.96 for MT of sinus measurements. The values obtained were tabulated; the mean average and respective standard deviations (SDs) were calculated for all parameters studied. The data analyses were performed by using the Statistical Package for the Social Sciences (SPSS), version 16.0 (SPSS Inc., Chicago, IL). Categorical variables were shown by n and % values and compared using The Mann Whitney U test. T-tests were used to compare measurements between left and right sides and between female and male patients. Statistical significance was determined at the level of *p* < 0.05.

## Results

The CBCT images of 410 maxillary sinuses and 1169 teeth (305 first premolars, 280 second premolars, 287 first molars and 297 second molars) of 205 individuals (49.3% males and 50.7% females), aged 16-77 years; (mean age: 38.8 ± 16.1) were examined.

- Prevalence and Pathologic Degree of MT

**Figure 1:**
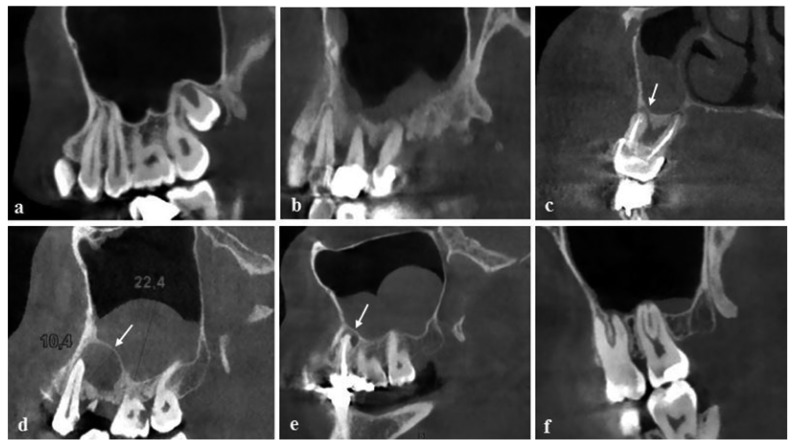
a-f.(a) Normal mucosa of the maxillary sinus, (b) severe periodontitis with exacerbating features and mucosal thickness, (c) mucosal thickness due to severe periodontal bone loss and apical periodontitis related with maxillary right first molar with root canal therapy , (d) mucosal thickness associated with the lesion in contact with the sinus floor, (e) tooth with root canal therapy and apical periodontitis with a well-defined radiolucent area and mucosal thickness, (f) sinus mucosal thickness due to root apex entered the sinus floor. Arrows indicate the periapical lesion.

Among 1169 examined teeth, 67.2% (786) of them presented with no maxillary sinusitis, no mucosal thickness or uniform mucosal thickness<2 mm (MT class 1,2) detected on the images,in either the left or right maxillary sinus (Fig. 1a). MT was observed in 383 (33.8%) of teeth which 54,9 % were 2-4 mm, 26,4% were 4-10 mm and 18,7% were more than 10 mm (MT class 3,4,5 respectively), ( Table 1). The mean mucosal thickness was 2,70 ± 3,73 (2.99 mm on the left side and 2.40 mm on the right side). In sinuses in which the thickness was more than 2 mm the mean mucosal thickness was 5.98 mm.

**Table 1:**
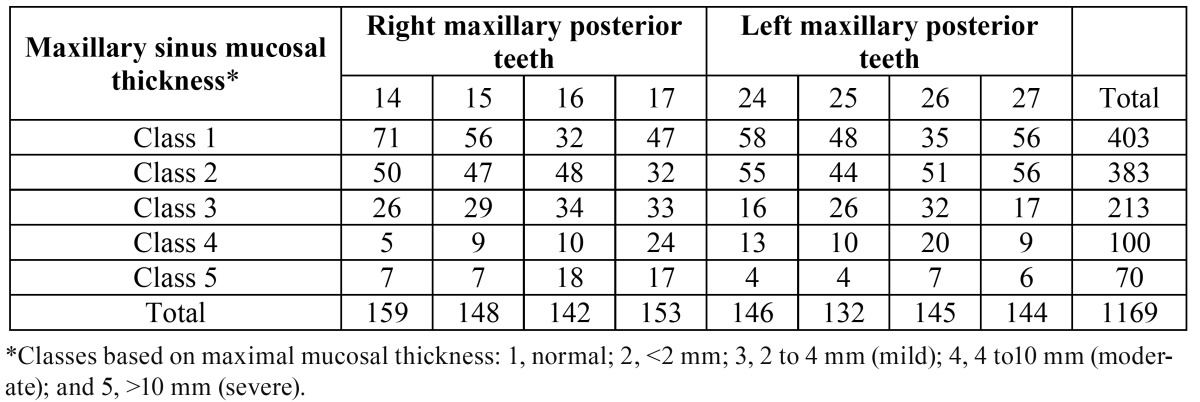
Distribution of Teeth due to the Maxillary Sinus Mucosal Thickness.

- Prevalence of Maxillary Sinus Mucosal thickness and Age

 The prevalence of maxillary sinus mucosal thickness was 21.4% (24/112) among the juvenile patients (≤18 years), 31.4% (84/267) among the young patients (19-25 years), 31.2% (125/400) among the adults (26-40 years), 51.2% (144/281) among the patients aged between 41 to 60 years, and 33% (36/109) among geriatric patients (>60 years) ( Table 2). Differences between age groups and the probability of presenting with sinus mucosal thickness were statistically significant (*p* =0 .002).

**Table 2:**
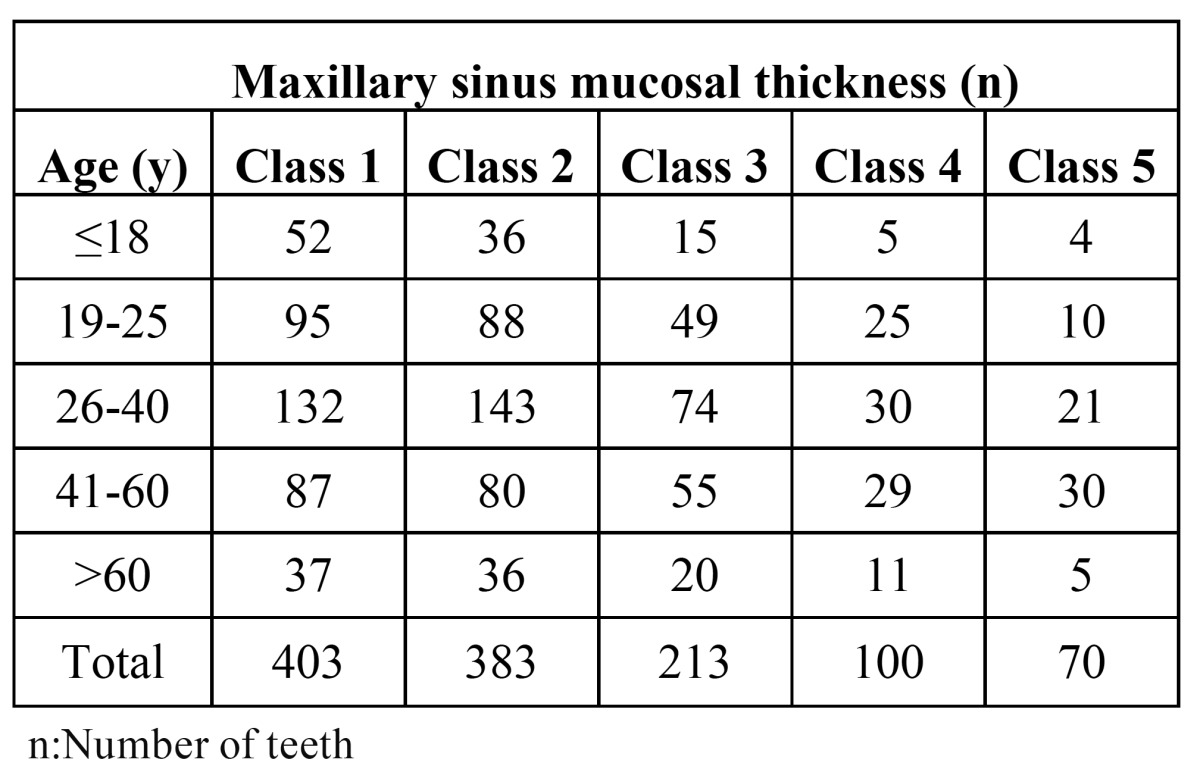
The Relationship between the Degree of Maxillary Sinus Mucosal Thickness and Age of Patient.

- Prevalence and Pathologic Degree of PBL and Periapical lesion in MT.

PBL was seen in 30 % of the patients, of whom 72% were mild, 18% were moderate and 10% were severe. The mean PBL was 0.89 mm ± 1. 53 mm. The independent t-test showed a higher prevalence of PBL in men than women (*p*< 0.05), whereas the difference between the mean bone loss was not significant (*p*> 0.05) except teeth numbered 17 and 24. Furthermore, the prevalence of sinus MT and the mean MT were significantly higher in men than women for teeth numbered 14, 15, 17 and 25 (*p*<0.05), ( Table 3). The Mann Whitney U test showed a significant difference between the mean sinus MT in individuals with PBL and without it (*p*< 0.01). Mean MT was 1.97 mm for teeth without PBL and 2.25 mm for teeth with PBL.There is a significant correlation between PBL and MT of maxillary sinus (r = 0.52, *p*<0.000), (Fig. 1b).

**Table 3:**
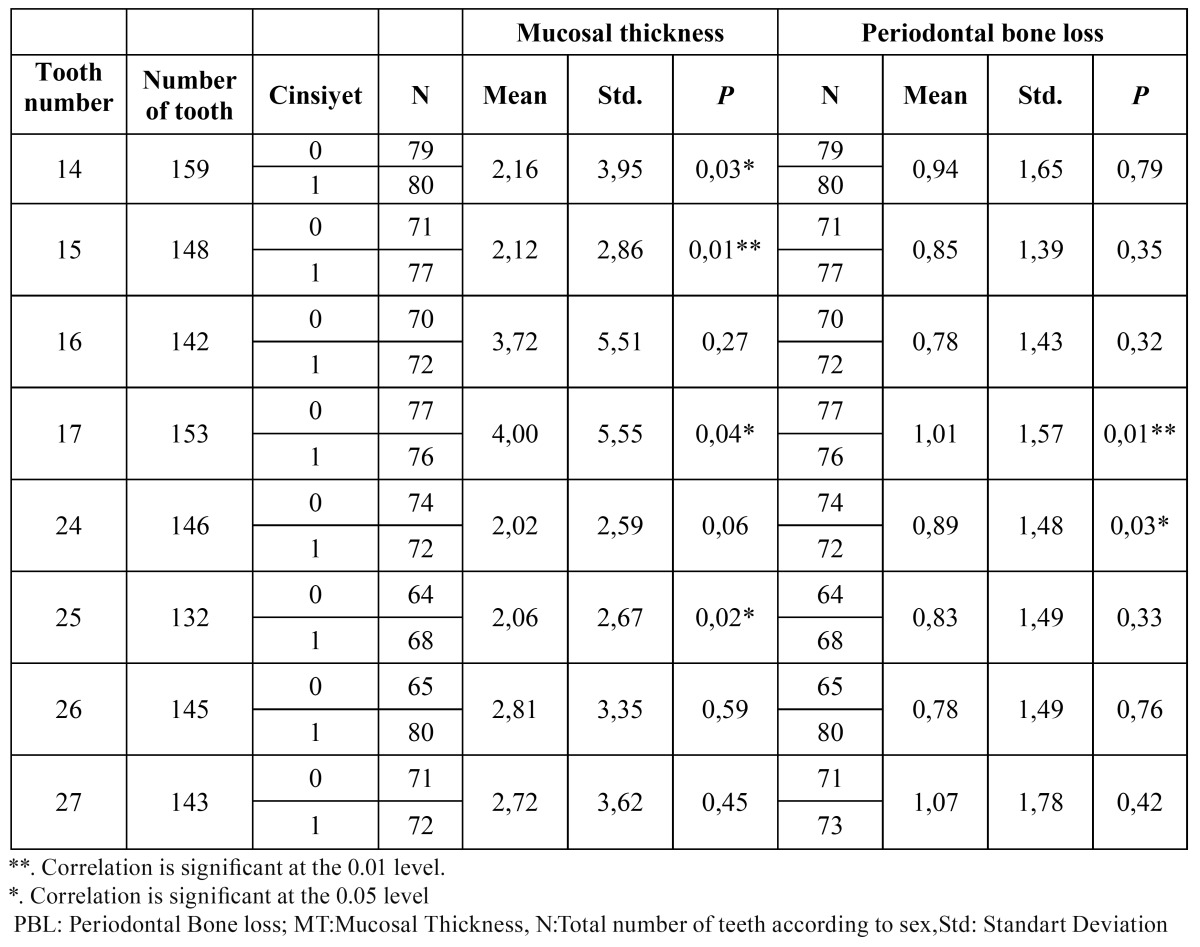
The mean Periodontal bone loss and Mucosal Thickness of sinus in males and females according to teeth.

Periapical lesions were found in 159 teeth and MT was seen in 100 (62.8%) of them. There were 41 teeth with periapical lesion that were associated with inferior wall of maxillary sinus (type 2,3,4,5) and the prevalence of maxillary sinus MT was 78% (32/41). Among the teeth with apical periodontitis, 41 were first premolar, 34 were second premolars, 48 were first molars and 36 were second molars. The possibility of maxillary sinus MT increased dramatically as the degree of apical periodontitis increased (Fig. 1c,d,e). The prevalence of MT for a patient with no apical periodontitis was 29.5% (298/1009) but increased to 58.8% (50/85), 70.5% (24/34,) 74.1% (23/31), and 33.3% (3/9) for those presenting with classes 2, 3, 4, and 5, respectively ( Table 4).

**Table 4:**
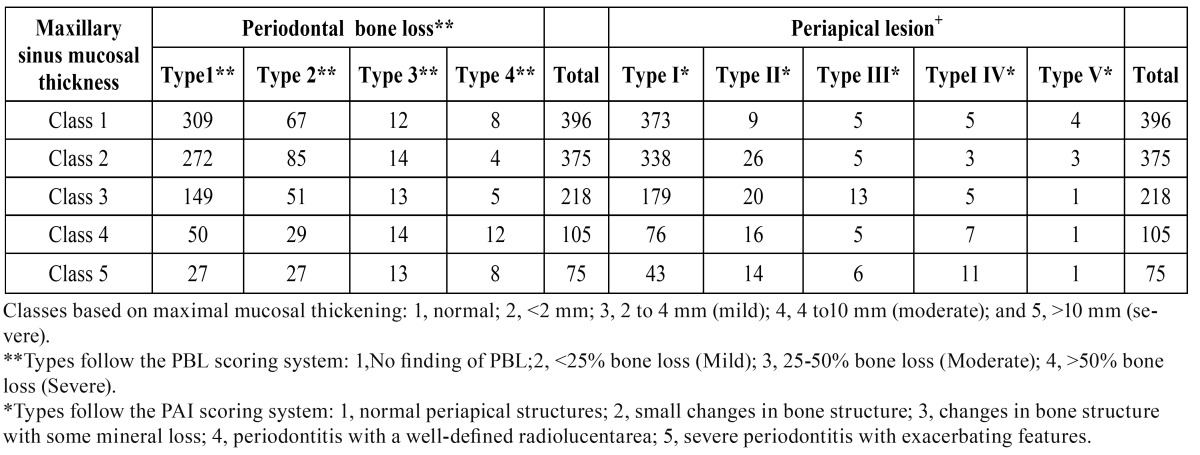
Association between Maxillary sinus Mucosal Thickness and Periapical Lesion Types and Degrees of Periodontal bone loss.

- The Anatomic Relationship between the Sinus Floor and Associated Teethand Prevalence of MT 

The most of patients, including all degrees of maxillary sinus MT, had a relationship between the root apex and sinus floor by a space (710/1169). The prevalence of maxillary sinus MT (a mean thickness of mucosa ≥2 mm) in these patients was 29.5% (210/710). In 314 teeth, the roots had contact with the sinus floor, and the prevalence of maxillary sinus MT was 34,3% (type 2, 108/314). In 145 teeth, the root apices projecting into the sinus cavity over the sinus floor, and the prevalence of maxillary sinus MT was 54.4% (type 3,4,5, 79/145) ( Table 5) (Fig. 1f). Among those teeth with periapical lesions, 118 had a space between the lesion and the sinus floor (prevalence of maxillary sinus mucosal thickness = 56.7%, 67/118). The lesion was found to be in contact with the sinus floor in 32 teeth (type 2, maxillary sinus MT = 71.8%, 23/32), whereas in the remaining 10 teeth with periapical lesions, the lesion entered the sinus floor (type 3,4,5, maxillary sinus MT = 90%, 9/10).

**Table 5:**
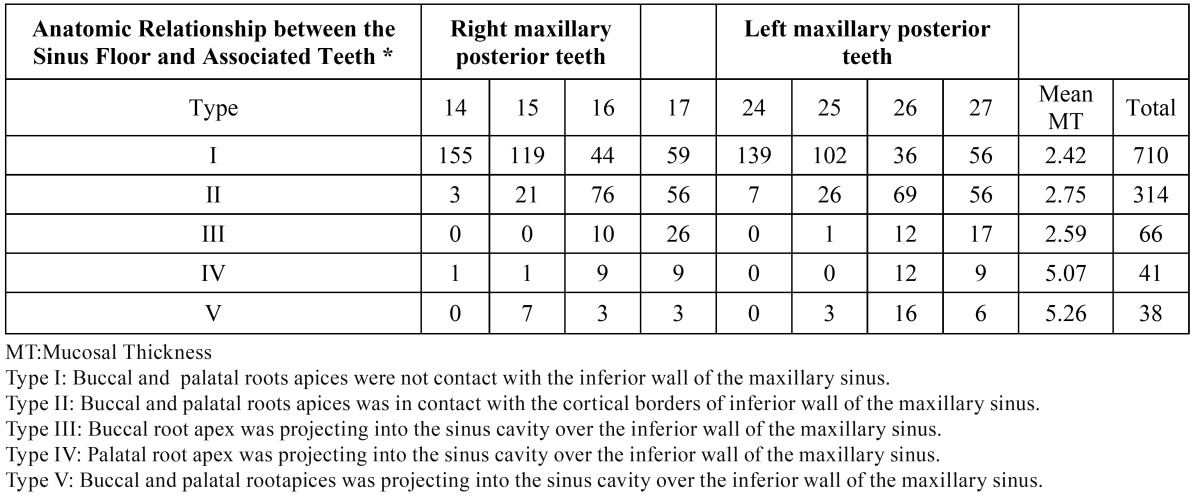
The Anatomic Relationship between the Sinus Floor and the Root Tips of Adjacent Teeth.

The teeth were classified into five groups to assess the effect of pulpoperiapical condition on the MT, the code number that is referred to each group shows the probable effect of pulpoperiapical condition of each tooth. The distribution of the teeth due to their pulpoperiapical conditions and the mean MT of the sinus in the different groups are shown in  Table 6. Bivariate correlation revealed a significant association between the pulpoperiapical condition and MT (r = 0.17, *p*=0.000).

**Table 6:**
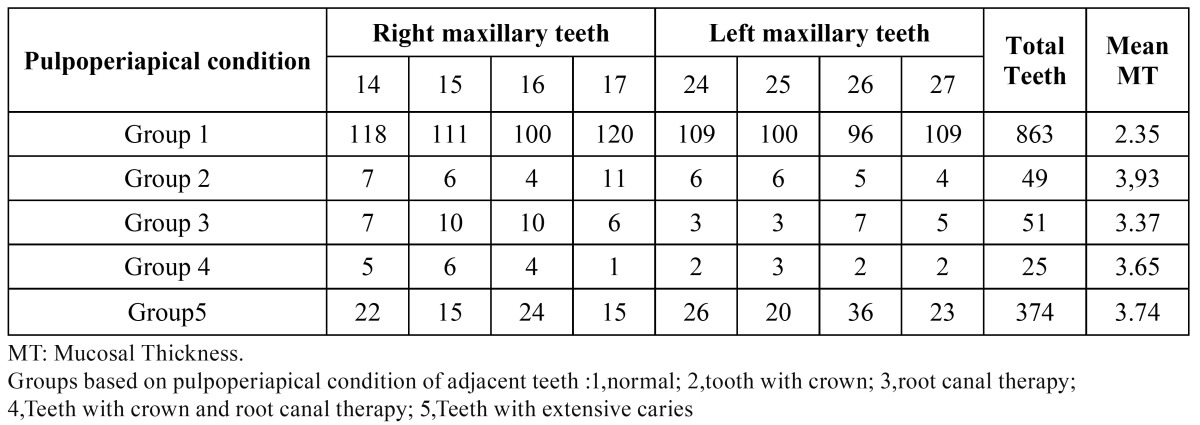
Distribution of teeth due to the Pulpoperiapical Condition.

## Discussion

The present study attempted to clarify the possible relationship between PBL, existence of apical lesion and MT of maxillary sinus. We assessed the degrees of PBL and severity of apical lesion as well as the distance between the sinus floor and the root apices using CBCT images.

CBCT is a technological invention without super imposition seen in conventional radiographic techniques. Anatomic structures are seen in clearer detail with the CBCT scanning. İt could be used in oral surgery, implant treatment planning, orthodontic evaluation, periodontal disease planning and apical periodontitis assessment (12). Some studies have reported that (13,14) conventional radiographic techniques have been used in which differentiation between maxillary sinus cysts and neoplasms, MT of infectious disease can be difficult. CBCT imaging was helpful in evaluating the preoperative and postoperative conditions of the maxillary sinuses. It was also was helpful in explaining the etiology and extent of the association between the dental pathology and the involved sinus (15,16).

The normal maxillary sinus MT is about 1 mm and homogenous thickness up to 2 mm is nonpathogenic. If mucosal thickness of >2 mm was evaluated as indication of MT (1). The prevalence of MT may change in accordance with the demarcation of normal MT. Savolainen *et al*. (4) defined abnormal thickness as a measure of more than 6 mm. Soikkonen and Ainamo did not define a baseline for maxillary sinus MT; their theory was the presence of diffuse radio-opacity along the maxillary sinus walls and they found the prevalence of 70% for sinus MT (13). Phothikhun *et al*. defined the MT when it was >1 mm and found MT in 42% of patients (17). Similar to our study, Janner *et al*. reported the existence of MT when it was more than 2 mm and found MT in 37% of patients (18). In the present study, the prevalence of MT was 33.8%. The MT was greater than 2 mm in most of the patients with maxillary sinusitis (19). Therefore, sinus MT more than 2 mm in depth was a significant indicator of maxillary sinusitis and was considered to be a pathologic form (3).

Several studies have reported varying prevalence rates of odontogenic maxillary sinusitis, ranging from 10% to 86% (1,20). One recent study reported that 98 of 135 maxillary sinus MT cases were tooth related, presenting with changes in the maxillary sinus floor (21). Periodontal disease (22,23), apical periodontitis (22), implant therapy (24) and tooth extraction (25) are thought to increase the risk of maxillary sinus MT. Marginal and apical periodontitis together constitute 83% of all dental causes (22).

Periodontitis is an inflammatory disease caused by specific microorganisms or groups of specific microorganisms, resulting in progressive destruction ofthe periodontal ligament and alveolar bone. It is considered as the second most common disease in the world after dental caries (1). In the U.S. has a prevalence of 30-50% of the population, but only about 10% had the severe type (26). Janner *et al*. revealed that there is a relationship between the maxillary sinus MT and PBL (18). Phothikhun *et al*. found that maxillary sinus MT was associated with PBL, especially in severe PBL (17). In the present study, the prevalence of PBL was found in 30% of patient, most ofthe patients had mild PBL and MT increased as the degree of PBL enlarged.

There was a significant correlation between MT and age (17).With age, most of individuals present with dental diseases, such as missing teeth, apical abscess or periodontitis, periodontal disease and other pathologic conditions and this have also triggered to increase the possibility of maxillary sinusitis (27). In the study of Yu Lu *et al*. (3) patients more than 60 years of age were found to be most likely to present with MT. But in our study, it was seen that maxillary sinus MT was higher (51.2%) among the patients aged between 41 to 60 years. In the study of Sheikhi *et al*. (1) a higher prevalence of MT and PBL was seen among males (*P* < 0.05), similar to the results of our study, the prevalence of PBL,sinus MT and the mean MT in men were observed higher than women.

Previous CBCT examinations have discovered a correlation between apical periodontitis and maxillary sinus MT (28). Using CBCT scanning, Maillet *et al*. (21) found that odontogenic sinusitis can be identified as localized MT of maxillary sinus related to dental infections (3). The complication of a periapical lesion related to a root apex that is in close proximity or penetrated into the sinus floor should be considered when a sinus infection is present specifically once the periapical disease reaches into the maxillary sinus, spreading and increasing the possibility of serious infections (3,29). The severity and incidence of maxillary sinus MT have been positively related with the degree of apical periodontitis (3). The local increased level of pathogenic bacteria and toxins and also inflammatory cytokines in apical lesions may directly infiltrate from the porous maxillary bone or they can reach indirectly through blood and lymph vessels. Both direct and indirect ways cause maxillary sinus MT (1,29,30). If the amount of bacteria and toxins increase, this results in an increase in the severity of periapical lesion and thus increases the possibility of maxillary sinus MT (3).

Yu Lu *et al*. (3) reported that, among teeth with periapical lesion, if there was a space between the lesion and the sinus floor MT was 77.3%, MT was 82.4% for teeth that the lesion was in contact with the sinus floor, 80.8% for teeth that the lesion entered the sinus floor. In the present study, the percentage of teeth that roots entered sinus floor was the mostly seen; root tips in relation to the sinus floor were as follows:54.4%> (Type 3,4,5) > 34,3% (Type 2)> 29.5% (Type 1). In teeth with periapical lesions, if there was a space between the lesion and the sinus floor MT was 56.7%, in teeth that the lesion was found to be in contact with the sinus floor MT was 71.8%, MT was 90% for teeth that the lesion entered the sinus floor.The locations of root tips or lesions were different and there were significant differences in the prevalence of maxillary sinus MT.

Pathologic dental findings and root canal treatments were also significantly associated with mucosal thickness (1). Resembling the present study, Nenzén and Welander (31) found that there was a relationship between root canal fillings and periapical lesions and maxillary sinus MT. Their results were in contrast with findings of Phothikhun *et al*. and Janner *et al*. (17,18). This dissimilarity can be due to the different classification of teeth. In present study, especially the teeth with crown and inadequate root canal therapy and extensive caries were associated with MT, pulpoperiapical condition is significantly related with MT (*p* = 0.000).

It is assessed that 10-12% of maxillary sinusitis cases have an odontogenic origin (28). In the cases of odontogenic sinusitis the most common pathogens are commonly anaerobic and which don’t respond to antibiotic therapies prescribed for common non-odontogenic pathogens and in the cases of non-odontogenic sinusitis pathogens are aerobic (32). Hence, diagnosing the exact source of infection is important for correct treatment planning. In the cases of maxillary sinus MT caused by periapical pathology or periodontal disease, endodontic and periodontal treatment to reduce the pathogens result in reduction of MT (20,31,33). In the present study, the majority of patients who were referred for CBCT are completely or partially edentulous patients that needed implant treatment or they had impacted teeth but not maxillary sinus problems. The absence of clinical investigation and exact history about the sinus problems were our limitations. There is a need for additional studies to clear up the accurate relationship between MT of maxillary sinus and possible underlying causes with regards to the clinical symptoms.

## Conclusions

Within the limitations of this study, it was revealed that there was a positive correlation between maxillary sinus MT and age of the patient and PBL. The anatomic relationship between root apices or periapical lesions and the maxillary sinus floor influenced the likelihood of maxillary sinus MT development.
